# Investigation of Structural and Luminescent Properties of Sol-Gel-Derived Cr‑Substituted Mg_3_Al_1−x_Cr_x_ Layered Double Hydroxides

**DOI:** 10.3390/molecules26071848

**Published:** 2021-03-25

**Authors:** Ligita Valeikiene, Inga Grigoraviciute-Puroniene, Arturas Katelnikovas, Aleksej Zarkov, Aivaras Kareiva

**Affiliations:** Institute of Chemistry, Vilnius University, Naugarduko 24, LT-03225 Vilnius, Lithuania; ligita.valeikiene@chgf.vu.lt (L.V.); inga.grigoraviciute@chf.vu.lt (I.G.-P.); arturas.katelnikovas@chf.vu.lt (A.K.); aivaras.kareiva@chgf.vu.lt (A.K.)

**Keywords:** layered double hydroxides, Mg_3_Al_1_, substitution effects, chromium, photoluminescence

## Abstract

In the present work, Cr-substituted Mg_3_Al_1−x_Cr_x_ layered double hydroxides (LDHs) were synthesised through the phase conversion of sol-gel-derived mixed-metal oxides in an aqueous medium. The chromium substitution level in the range of 1 to 25 mol% was investigated. It was demonstrated that all synthesised specimens were single-phase LDHs. The results of elemental analysis confirmed that the suggested synthetic sol-gel chemistry approach is suitable for the preparation of LDHs with a highly controllable chemical composition. The surface microstructure of sol-gel-derived Mg_3_Al_1−x_Cr_x_ LDHs does not depend on the chromium substitution level. The formation of plate-like agglomerated particles, which consist of hexagonally shaped nanocrystallites varying in size from approximately 200 to 300 nm, was observed. Optical properties of the synthesised Mg_3_Al_1−x_Cr_x_ LDHs were investigated by means of photoluminescence. All Cr-containing powders exhibited characteristic emission in the red region of the visible spectrum. The strongest emission was observed for the sample doped with 5 mol% Cr^3+^ ions. However, the emission intensity of samples doped with 1–10 mol% Cr^3+^ ions was relatively similar. A further increase in the Cr^3+^ ion concentration to 25 mol% resulted in severe concentration quenching.

## 1. Introduction

Layered double hydroxides (LDHs) are compounds composed of positively charged brucite-like layers with an interlayer gallery containing charge-compensating anions and water molecules. The metal cations occupy the centres of shared oxygen octahedra whose vertices contain hydroxide ions that connect to form infinite two-dimensional sheets [1,2,3]. The chemical formula of layered double hydroxides is expressed as (M^2+^_x_M^3+^_1−x_(OH)_2x_A·zH_2_O), where M^2+^ and M^3+^ are divalent and trivalent metal ions, respectively, and A^−^ is an intercalate anion that compensates the positive charge created by the partial substitution of M^2+^ by M^3+^^+^ ions in brucite-type layers [1,2,3]. LDH structures containing Mg^2+^, Zn^2+^, Co^2+^, Ni^2+^, Cu^2+^ or Mn^2+ +^ ion as divalent cations and Al^3+^, Cr^3+^, Co^3+^, Fe^3+^, V^3+^, Y^3+^ or Mn^3+ +^ ion as trivalent ones are known. LDHs with many different anionic species have been reported: both inorganic anions (carbonate, chloride, nitrate, sulphate, molybdate, phosphate, etc.) and organic anions (terephthalate, acrylate, lactate, etc.). LDHs are widely used as adsorbents, catalyst support precursors, anion exchangers, acid residue scavengers, flame retardants, osmosis membranes, sensors and others [4,5,6,7,8,9,10,11,12]. After calcination at temperatures from 300 to 600 °C, LDHs are converted to mixed-metal oxides (MMOs) with a high specific surface area and basic properties. The ability of MMOs to recover the original layered structure is a property known as the memory effect [4,9]. When MMOs are immersed in an aqueous solution that contains some anions, the layered structure can be recovered with those anions intercalated into the interlayer.

As mentioned before, chromium-containing LDHs have also been synthesised, and these compounds show interesting chemical and physical properties. For example, chromium-containing Mg-Cr and Ni-Cr LDHs have already been used as precursors to synthesise pillared derivatives with decavanadate anions without the use of any preswelling agent [13]. The decomposition products of these LDHs were successfully applied as adsorbents [14]. Zn-Cr LDH samples were synthesised using the classical co-precipitation approach from aqueous solution [15]. The adsorption capacities of the synthesised samples were investigated performing experiments with Indigo Carmine as the model of organic pollutant. Moreover, Zn-Cr and Zn-Cr LDHs, intercalated with the Keggin ion, showed a high adsorption capacity for effective removal of iron (II) from aqueous solution [16].

M(II)-Cr (M = Co, Ni, Cu and Zn) LDHs also show interesting photocatalytic properties and have been used for photocatalytic degradation of organic pollutants [17,18]. The decomposition products of different LDHs containing chromium have also been tested as catalysts [19,20]. It was also demonstrated that Mg-Cr LDHs containing composites can be used for the preparation of coatings that show optimal corrosion protection [21].

Considerable attention has been focused on incorporating different elements into LDH host layers to develop new functional materials that resemble designed optical properties. The effect of sensitising anions [22], multiwavelength luminescence [23], the size effect [24] and the influence of carboxylate [25] and terephtalate [26] on the optical properties of lanthanide-containing LDHs was investigated. Moreover, series of LDHs and mixed-metal oxides with different lanthanide cations were also prepared [27]. Smalenskaite et al. focused on the characterisation of LDHs doped with Tb^3+^ [28], Nd^3+^ [29] and Eu^3+^ [30]. Chromium was also used as a substituent in different LDHs for various reasons. For instance, Cr-substituted Mg-Al LDHs were successfully used as a catalyst in aldose-ketose isomerisation processes [31]. Mixed oxides, obtained by thermal decomposition of Cr-substituted LDHs, were studied in the reaction of hydrocarbon steam reforming for the production of hydrogen [32]. Rodriguez-Rivas et al. [33] recently suggested to use Cr-substituted Zn-Al LDHs as UV–VIS light photocatalysts for NO gas removal from the urban environment. Finally, the spectral emission of natural LDHs was studied by cathodo- and photoluminescence (PL) techniques [34]. It was concluded that the presence of Cr^3+^ ion activators induces characteristic PL emission peaks in the red-infrared region at 681, 688 and 696 nm linked to ^2^E→^4^A_2_ transitions. However, the luminescent properties of Cr-doped or Cr-substituted Mg-Al LDHs have not been investigated so far to the best of our knowledge.

A sol-gel method based on in situ generation of mixed-metal chelates by complexing metal ions with various complexing agents in aqueous media was successfully used to prepare different monophasic inorganic compounds and nanostructures, such as biomaterials [35] and magnetic [36] and optical [37,38] materials. Previously, for the preparation of Mg-Cr and related LDHs, the non-aqueous sol-gel chemistry approach has been explored using metal alkoxides as starting materials [39,40]. We recently developed the indirect aqueous sol-gel method for the preparation of LDHs. We demonstrated that the synthesis of LDHs using this simple and environmentally benign method ensures the formation of homogeneous, monophasic samples having particles with a narrow size distribution [4,9,11,41]. It is well known that application of a synthesis method also depends on the chemical composition of the compound to be synthesised. The aim of this work was to synthesise Mg_3_Al_1‑x_Cr_x_ LDH samples for the first time using an aqueous sol-gel method and to investigate the effect of Cr^3+^ substitution on phase purity, morphological and luminescent properties of obtained end products. The luminescent properties of sol-gel-derived Cr-substituted Mg-Al LDHs was also investigated for the first time to the best of our knowledge.

## 2. Results and Discussion

**Figure 1:**
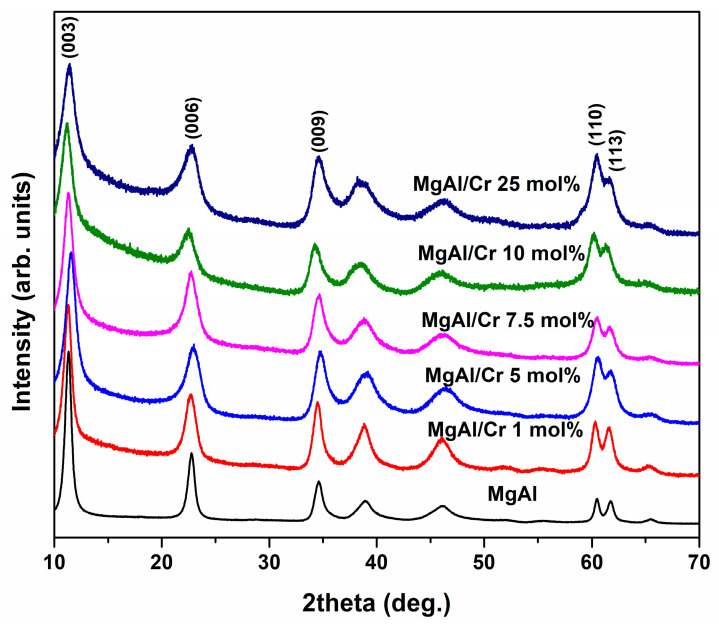
X-ray diffraction (XRD) patterns of Cr-substituted Mg_3_Al_1−x_Cr_x_ LDHs.

The XRD patterns of Mg_3_Al_1_ and Mg_3_Al_1−x_Cr_x_ LDHs with different chromium substitution levels (1–25 mol%) are shown in Figure 1. Characteristic diffraction peaks for the LDH structure were observed at 2θ angles at ca. 11.5° (003), 23° (006) and 34.5° (009). Besides, two additional LDH peaks were clearly displayed at about 60.5° and 61.5°, which correspond to reflections from the (110) and (113) planes [4]. No diffraction lines attributable to the side phases could be observed in the XRD patterns of sol-gel-derived LDH samples. Less important reflections (015) and (018) visible at about 38–39° and 47–49°, respectively, were also attributed to the LDH phase. It is interesting to note that only 9 mol% of chromium was introduced to Mg_3_Al_1−x_Cr_x_ LDHs when the impregnation method (adsorption) was used for the preparation [31]. It was also observed that aluminium substitution by chromium reduces the crystallinity of LDHs synthesised by the co-precipitation method [32].

The calculated values of d-spacing and lattice parameters of Mg_3_Al_1−x_Cr_x_ LDHs are summarised in Table 1. As seen, both *d* values and lattice parameters slightly increased with increasing amounts of chromium in the LDH structure. This was expected, since a smaller Al^3+^ ion (0.535 Å, CN = 6) was monotonically replaced by a larger Cr^3+^ ion (0.615 Å, CN = 6) [42]. The calculated crystallite sizes of the Mg_3_Al_1_ and Mg_3_Al_1−x_Cr_x_ LDHs were independent of the amount of chromium and varied in the range of 21.8–26.7 nm.

As seen from Table 1, the calculated lattice parameters also monotonically increased with increasing amounts of chromium: from 3.0374 to 3.1158 Å (a parameter) and from 22.8100 to 23.6586 Å (c parameter) with increasing amounts of chromium from 0% to 25%, respectively.

**Figure 2:**
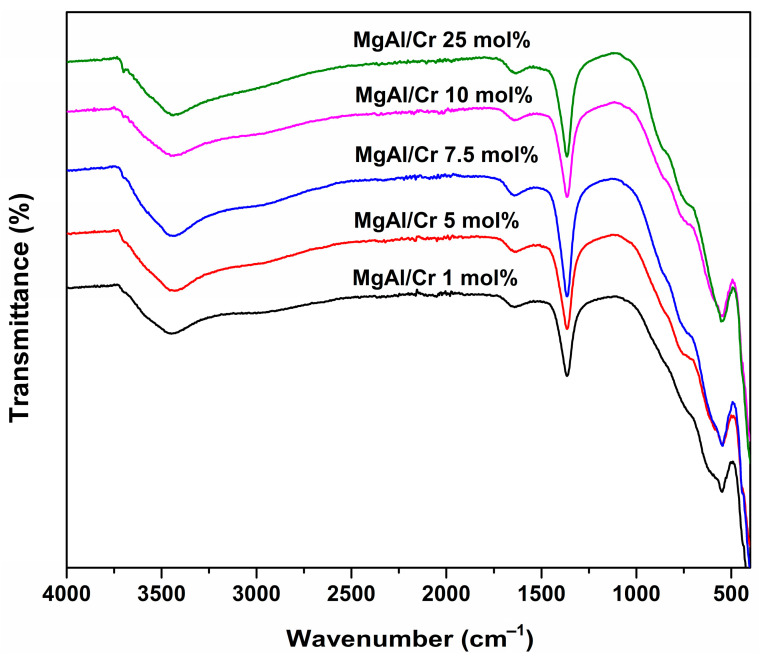
The Fourier transform infrared (FT-IR) spectra of Cr-substituted Mg_3_Al_1−x_Cr_x_ LDHs.

The FT-IR spectra of Cr-substituted Mg_3_Al_1−x_Cr_x_ LDHs are presented in Figure 2.

Figure 2 clearly shows that all IR spectra were almost identical, i.e., independent of the chromium substitution level. The broad absorption bands visible in the range of 3650–3250 cm^−1^ could be attributed to the stretching vibrations of hydroxyl (-OH) groups and water molecules located between the layers in the LDH crystal structure [43]. The low-intensity bands observed at 1630–1600 cm^−1^ confirmed the presence of adsorbed molecular water on the surface of sol-gel-derived LDHs. The intense absorption bands located at about 1350 cm^−1^ could be attributed to the asymmetric vibration modes of carbonate ions (CO_3_^2−^) [43]. Finally, the pronounced absorption bands observed at about 600 and 480 cm^−1^ were attributable to metal–oxygen vibrations [11,43]. These results are in a good agreement with X-ray diffraction (XRD) analysis data confirming the formation of Cr-substituted Mg_3_Al_1−x_Cr_x_ LDHs. Similar results were observed during FT-IR studies of sol-gel-derived Mg_3_Al_1−x_Cr_x_ [4] or Mg_2-x_M_x_/Al_1_ (M = Ca, Sr, Ba) [41] LDH samples.

**Figure 3:**
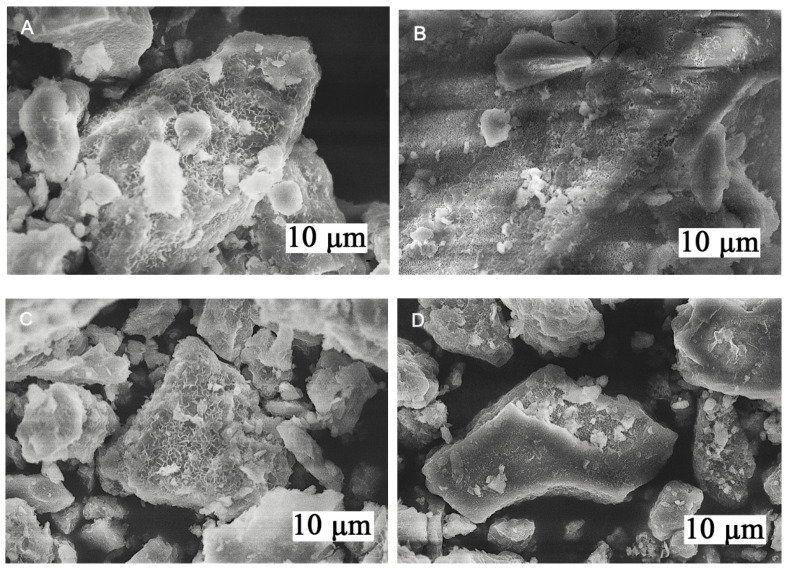
Scanning electron microscope (SEM) micrographs of Cr-substituted Mg_3_Al_1−x_Cr_x_ LDHs. The amount of Cr in mol%: (**A**) 1, (**B**) 5, (**C**) 10 and (**D**) 25. The scale bars are the lengths of rectangles.

SEM micrographs of Cr-substituted Mg_3_Al_1−x_Cr_x_ LDHs prepared by the sol-gel method are depicted in Figure 3. Again, the surface microstructure of sol-gel-derived Mg_3_Al_1−x_Cr_x_ LDHs did not depend on the chromium substitution level. Moreover, the characteristic features of LDHs could be observed in all presented SEM micrographs [4,9,11,28,29,30,41,43]. The formation of plate-like agglomerated particles, which consist of hexagonally shaped nanocrystallites varying in size from approximately 200 to 300 nm, was observed.

Elemental analysis of synthesised Cr-substituted Mg_3_Al_1−x_Cr_x_ LDHs was performed prior to investigation of luminescent properties. ICP-OES and EDX were used for the determination of chromium, magnesium and aluminium in the synthesised LDH samples. The results of elemental analysis are presented in Table 2. The summarised results indicate that the molar ratios of Mg, Al and introduced amounts of Cr are in a good agreement with those obtained by both analysis methods and coincide with nominal ones.

Thus, this study for the first time demonstrated that the previously developed sol-gel technique is suitable for the preparation of Cr-substituted LDHs.

**Figure 4:**
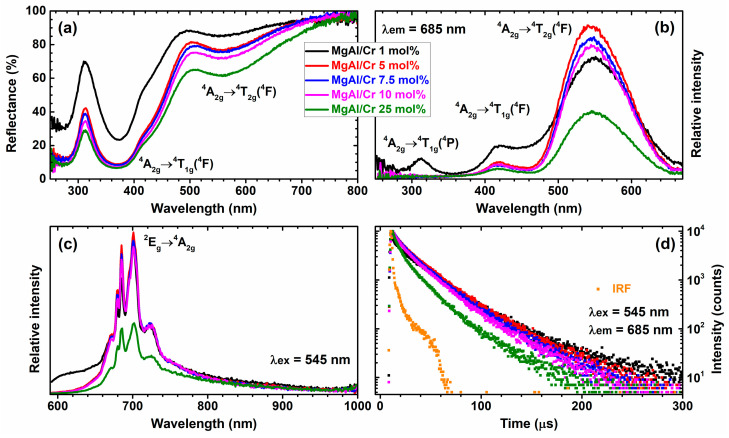
Reflection (**a**), excitation (**b**), emission (**c**) and photoluminescence (PL) emission decay curves (**d**) of Mg_3_Al_1‑x_Cr_x_ LDHs as a function of Cr^3+^ concentration. Instrument response function (IRF) in section (**d**) stands for instrument response function.

The reflection spectra of Mg_3_Al_1−x_Cr_x_ LDHs as a function of Cr^3+^ concentration are given in Figure 4a. All the spectra contained two broad absorption bands with maxima at ca. 550 and 380 nm. These bands could be assigned to the Cr^3+^ optical transitions of ^4^A_2g_→^4^T_2g_(^4^F) and ^4^A_2g_→^4^T_1g_(^4^F), respectively [44,45]. The increase in the Cr^3+^ concentration in the samples resulted in increased absorption.

Excitation spectra (λ_em_ = 685 nm) of the synthesised specimens are shown in Figure 4b. Similar to the reflection spectra, there were several broad bands in the excitation spectra as well. One band possessed the maximum at ca. 545 nm and was attributed to the ^4^A_2g_→^4^T_2g_(^4^F) optical transition, whereas the other band peaked at ca. 420 nm and could be assigned to the ^4^A_2g_→^4^T_1g_(^4^F) optical transition of Cr^3+^ ions. It is interesting to note that excitation spectrum of the 1% Cr^3+^-doped sample (Mg-Al/Cr 1 mol%) contained the third band at ca. 310 nm. This band arose from the ^4^A_2g_→^4^T_1g_(^4^P) transitions of Cr^3+^ ions.

Emission spectra of Mg_3_Al_1−x_Cr_x_ LDHs samples are depicted in Figure 4c. There were several relatively sharp emission lines in the range of 660–740 nm that could be assigned to the ^2^E_g_→^4^A_2g_ optical transition of Cr^3+^ ions. The strongest emission was observed for the sample doped with 5% Cr^3+^ ions. Surprisingly, the highest intensity of the ^5^D_0_→^7^F_2_ transition for Mg_3_Al_1−x_Ce_x_ LDHs was observed for the specimen with 0.05 mol% of Ce^3+^ [4]. It turned out that the emission intensity decreased with increasing concentration of Ce^3+^ up to 1 mol%. In the case of Mg_3_Al_1−x_Nd_x_ LDHs, the luminescence of Nd^3+^ was observed only with intercalation of terephthalate in the interlayer spacing [29].

However, the emission intensity of samples doped with 1–10% Cr^3+^ was relatively similar. A further increase in the Cr^3+^ concentration to 25% resulted in severe concentration quenching. These results correlate with Mg_3_Al_1‑x_Cr_x_ LDHs’ PL decay curves given in Figure 4d. The PL decay curves of samples doped with 1% to 10% Cr^3+^ were packed close together, indicating similar PL lifetime values. This was not, however, true for the PL decay curve of samples doped with 25% Cr^3+^. This curve was much steeper, indicating lower PL lifetime values. The effective PL lifetime values were calculated according to the following equation [46]:

(1) $$\tau_{eff}=\frac{\int_{0}^{\infty }I(t)tdt}{\int_{0}^{\infty }I(t)dt}$$

Here, *I(t)* is the PL intensity at the given time *t*. The obtained effective PL lifetime values (*τ_eff_*) for 1%, 5%, 7.5%, 10% and 25% Cr^3+^-doped samples were 23.0, 22.4, 20.6, 19.2 and 13.5 μs, respectively. Obviously, the *τ_eff_* values gradually decreased with increasing Cr^3+^ concentration, which indicates that the internal efficiency of Cr^3+^ ions decreases as well. The results obtained clearly show that Mg_3_Al_1−x_Cr_x_ LDHs are suitable for electro-optical application. For instance, the material can be potentially applied as a red light-emitting diode (LED) [47]. Besides, these compounds show potential as imaging systems for biomedical applications.

## 3. Materials and Methods

### 3.1. Materials

For the synthesis of Cr-substituted Mg_3_Al_1−x_Cr_x_ LDH samples, aluminium nitrate nonahydrate (Al(NO_3_)_3_·9H_2_O, 98.5%, Chempur, Plymouth, MI, USA), magnesium nitrate hexahydrate (Mg(NO_3_)_2_·6H_2_O), 99.0%, Chempur, Plymouth, MI, USA) and chromium nitrate nonahydrate (Cr(NO_3_)_3_∙9H_2_O, 99.0%, Aldrich, Darmstadt, Germany) were used as starting materials. Citric acid (C_6_H_8_O_7_·H_2_O, 99.5%, Chempur, Plymouth, MI, USA) and 1,2-ethanediol (C_2_H_6_O_2_, 99.8%, Chempur, Plymouth, MI, USA) were used as complexing agents. The reconstruction of MMO powders to LDHs was performed in distilled water.

### 3.2. Synthesis

**Figure 5:**
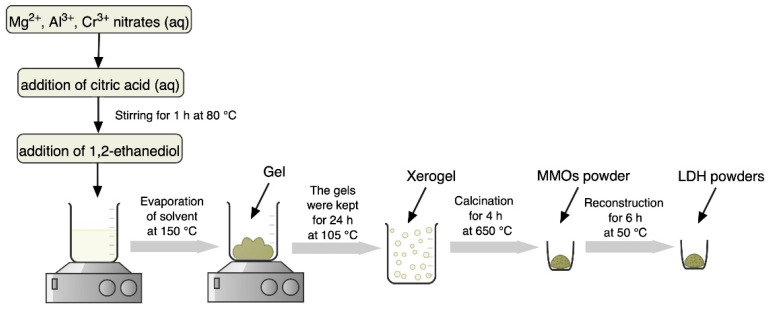
Schematic diagram of the sol-gel preparation of Mg_3_Al_1−x_Cr_x_ layered double hydroxides (LDHs).

Cr-substituted Mg_3_Al_1−x_Cr_x_ LDH samples (x = 0.0, 0.01, 0.05, 0.075, 0.1 and 0.25) were synthesised using Al(NO_3_)_3_·9H_2_O, Mg(NO_3_)_2_·6H_2_O and Cr(NO_3_)_3_∙9H_2_O. The aqueous solution of starting materials was mixed with 0.2 M solution of citric acid and 2 mL of 1,2-ethanediol under continuous stirring at 80 °C for 1 h. Next, the temperature of the magnetic stirrer was raised to 150 °C until complete evaporation of the solvent. The obtained gels were kept at 105 °C for 24 h. Mixed-metal oxides (MMOs) were obtained by calcination of the gels at 650 °C for 4 h. Mg_3_Al_1−x_Cr_x_ LDHs were obtained by reconstruction of MMOs powders in distilled water at 50 °C for 6 h under stirring. The schematic representation of the sol-gel processing is illustrated in Figure 5.

### 3.3. Characterisation

For characterisation of the phase purity of the synthesised materials, powder X-ray diffraction (XRD) analysis was performed in the 2θ range from 10° to 70° (step size of 0.02°) with a scanning speed of 2°/min using a MiniFlex II diffractometer (Rigaku, the Woodlands, TX, USA) (Cu Kα radiation). The size of crystallites was calculated by the Scherrer equation:

(2) $$\tau =\frac{0.9\lambda }{Bcos\theta }$$

where *τ* is the mean crystallite size, *λ* is the X-ray wavelength, *B* is the line broadening of full width at half maximum (FWHM) intensity and *θ* is the Bragg angle. Fourier transform infrared (FT-IR) spectra were collected using an Alpha spectrometer (Bruker, Inc., Billerica, MA, USA). All spectra were recorded at ambient temperature in the range of 4000–480 cm^−1^. The operational mode was transmittance (%). The morphology of synthesised LDH samples was investigated using a scanning electron microscope (SEM) (Hitachi SU-70, Tokyo, Japan). The energy-dispersive X-ray (EDX) analysis of the specimens was performed using a SEM Hitachi TM 3000. The reflection, photoluminescence emission (PL) and excitation (PLE) spectra were recorded on an Edinburg Instruments FLS980 modular spectrometer (Edinburgh Instruments Ltd., Kirkton Campus, Livingstone UK). The spectrometer was equipped with a 450 W Xe lamp (Edinburgh Instruments Ltd., Kirkton Campus, Livingstone, UK) as an excitation source, a cooled (−20 °C) photomultiplier (Hamamatsu, Iwata, Japan) (R928P) and double-grating excitation and emission monochromators. Both excitation and emission spectra were corrected for the instrument spectral response. Elemental analysis of the synthesised samples was performed by inductively coupled plasma–optical emission spectrometry (ICP-OES) using an Optima 7000DV spectrometer (Perkin-Elmer, Waltham, MA, USA). The samples were dissolved in 5% nitric acid (HNO_3_, Rotipuran^®^ Supra 69%, Carl Roth) and diluted to an appropriate volume. Calibration solutions were prepared by an appropriate dilution of stock standard solutions (single-element ICP standards 1000 mg/L, Carl Roth, Karlsruhe, Germany).

## 4. Conclusions

In conclusion, Mg_3_Al_1−x_Cr_x_ layered double hydroxides (LDHs) within the substitution range of chromium from 1 to 25 mol% were successfully synthesised for the first time by an aqueous sol-gel processing route. XRD analysis and FT-IR spectroscopy results confirmed that the synthesised Mg_3_Al_1−x_Cr_x_ LDHs were predominant crystalline phases in the end products. The calculated values of d-spacing and lattice parameters of Mg_3_Al_1−x_Cr_x_ LDHs slightly increased with increasing amounts of chromium. The sol-gel-derived Mg_3_Al_1−x_Cr_x_ LDHs consisted of characteristic hexagonally shaped nanoparticles 200–300 nm in size regardless of the chromium substitution level. All Cr-containing powders exhibited characteristic emission in the red region of the visible spectrum. The major emission lines of Mg_3_Al_1−x_Cr_x_ LDHs excited at 545 nm peaked in the red spectral region at 680–695 nm, originating from the ^2^E_g_→^4^A_2_ transition. The highest intensity of emission was observed for the Mg_3_Al_1−x_Cr_x_ LDH specimen containing 5% of Cr^3+^. With further increasing the chromium content up to 25%, concentration quenching was observed. Concentration quenching was also confirmed by the calculated PL effective lifetime values (*τ_eff_*), which decreased from 22.4 μs for the 5% Cr^3+^-doped sample to 13.5 μs for the 25% Cr^3+^-doped sample.

## Figures and Tables

**Table 1:** The values of d-spacing and lattice parameters of Mg_3_Al_1−x_Cr_x_ layered double hydroxides (LDHs) calculated by the Le Bail method.

Sample	d (003), Å	d (006), Å	d (110), Å	Lattice Parameters, Å	
				a	c
Mg-Al	7.6033	3.8017	1.5187	3.0374	22.8100
Mg-Al/Cr 1 mol%	7.7470	3.8735	1.5242	3.0484	23.2408
Mg-Al/Cr 5 mol%	7.7748	3.8876	1.5276	3.0551	23.3248
Mg-Al/Cr 7.5 mol%	7.8028	3.9015	1.5314	3.0627	23.4094
Mg-Al/Cr 10 mol%	7.8373	3.9187	1.5367	3.0733	23.5122
Mg-Al/Cr 25 mol%	7.8774	3.9475	1.5579	3.1158	23.6586

**Table 2:** Inductively coupled plasma–optical emission spectrometry (ICP-OES) and the energy-dispersive X-ray (EDX) analysis results of elemental analysis of synthesised Mg_3_Al_1−x_Cr_x_ LDHs (n is mole).

Sample	ICP-OES		EDX	
	n(Cr), %	n(Mg):n(Al + Cr)	n(Cr), %	n(Mg):n(Al + Cr)
Mg-Al/Cr 1 mol%	1.12	3:0.994	1.43	3:0.993
Mg-Al/Cr 5 mol%	5.39	3:0.990	7.22	3:1.06
Mg-Al/Cr 7.5 mol%	7.91	3:0.988	7.77	3:1.02
Mg-Al/Cr 10 mol%	10.5	3:0.997	12.9	3:0.875
Mg-Al/Cr 25 mol%	25.8	3:1.01	26.3	3:1.02

## Data Availability

Data is contained within the article.

## Sample Availability

Samples of the compounds are available from the authors.

## References

[1] Mishra G., Dash B., Pandey S. (2018). Layered double hydroxides: A brief review from fundamentals to application as evolving biomaterials. Appl. Clay Sci..

[2] Arrabito G., Bonasera A., Prestopino G., Orsini A., Mattoccia A., Martinelli E., Pignataro B., Medaglia P.G. (2019). Layered Double Hydroxides: A Toolbox for Chemistry and Biology. Crystals.

[3] Mohapatra L., Parida K. (2016). A review on the recent progress, challenges and perspective of layered double hydroxides as promising photocatalysts. J. Mater. Chem. A.

[4] Smalenskaite A., Vieira D.E.L., Salak A.N., Ferreira M.G.S., Katelnikovas A., Kareiva A. (2017). A comparative study of co-precipitation and sol-gel synthetic approaches to fabricate cerium-substituted Mg-Al layered double hydroxides with luminescence properties. Appl. Clay Sci..

[5] Modrogan C., Caprarescu S., Dancila A.M., Orbulet O.D., Vasile E., Purcar V. (2020). Mixed oxide-layered double hydroxides materials: Synthesis, characterization and efficient application for Mn^2+^ removal from synthetic wastewater. Materials.

[6] Shi M., Zhao Z., Song Y., Xu M., Li J., Yao L. (2020). A novel heat-treated humic acid/MgAl-layered double hydroxide composite for efficient removal of cadmium: Fabrication, performance and mechanisms. Appl. Clay Sci..

[7] Zhang S., Kano N., Mishima K., Okawa H. (2019). Adsorption and Desorption Mechanisms of Rare Earth Elements (REEs) by Layered Double Hydroxide (LDH) Modified with Chelating Agents. Appl. Sci..

[8] Soliman E.R., Kotp Y.H., Souaya E.R., Guindy K.A., Ibrahim R.G.M. (2019). Development the sorption behavior of nanocomposite Mg/Al LDH by chelating with different monomers. Compos. Part B Eng..

[9] Sokol D., Salak A.N., Ferreira M.G.S., Beganskiene A., Kareiva A. (2018). Bi-substituted Mg3Al–CO3 layered double hydroxides. J. Sol-Gel Sci. Technol..

[10] Vieira D.E.L., Sokol D., Smalenskaite A., Kareiva A., Ferreira M.G.S., Vieira J.M., Salak A.N. (2019). Cast iron corrosion protection with chemically modified Mg-Al layered double hydroxides synthesized using a novel approach. Surf. Coat. Technol..

[11] Valeikiene L., Paitian R., Grigoraviciute-Puroniene I., Ishikawa K., Kareiva A. (2019). Transition metal substitution effects in sol-gel derived Mg_3-x_M_x_/Al_1_ (M = Mn, Co, Ni, Cu, Zn) layered double hydroxides. Mater. Chem. Phys..

[12] Szabados M., Adél Ádám A., Traj P., Muráth S., Baán K., Bélteky P., Kónya Z., Kukovecz Á., Sipos P., Pálinkó I. (2020). Mechanochemical and wet chemical syntheses of CaIn-layered double hydroxide and its performance in a transesterification reaction compared to those of other Ca2M(III) hydrocalumites (M: Al, Sc, V, Cr, Fe, Ga) and Mg(II)-, Ni(II)-, Co(II)- or Zn(II)-based hydrotalcites. J. Catal..

[13] Kooli F., Rives V., Ulibarri M.A. (1995). Preparation and Study of Decavanadate-Pillared Hydrotalcite-like Anionic Clays Containing Transition Metal Cations in the Layers. 2. Samples containing Magnesium-Chromium and Nickel-Chromium. Inorg. Chem..

[14] Kooli F., Martín C., Rives V. (1997). FT-IR Spectroscopy Study of Surface Acidity and 2-Propanol Decomposition on Mixed Oxides Obtained upon Calcination of Layered Double Hydroxides. Langmuir.

[15] Bouteraa S., Saiah F.B.D., Hamouda S., Bettahar N. (2020). Zn-M-CO3 Layered Double Hydroxides (M=Fe, Cr, or Al): Synthesis, Characterization, and Removal of Aqueous Indigo Carmine. Bull. Chem. React. Eng. Catal..

[16] Oktrianty M., Palapa N.R., Mohadi R., Lesbani A. (2020). Effective Removal of Iron (II) from Aqueous Solution by Adsorption using Zn/Cr Layered Double Hydroxides Intercalated with Keggin Ion. J. Ecolog. Eng..

[17] Mohapatra L., Parida K.M. (2012). Zn–Cr layered double hydroxide: Visible light responsive photocatalyst for photocatalytic degradation of organic pollutants. Sep. Purif. Technol..

[18] Baliarsingh N., Parida K.M., Pradhan G.C. (2014). Effects of Co, Ni, Cu, and Zn on Photophysical and Photocatalytic Properties of Carbonate Intercalated MII/Cr LDHs for Enhanced Photodegradation of Methyl Orange. Ind. Eng. Chem. Res..

[19] Tsyganok A.I., Inaba M., Tsunoda T., Uchida K., Suzuki K., Takehira K., Hayakawa T. (2005). Rational design of Mg–Al mixed oxide-supported bimetallic catalysts for dry reforming of methane. Appl. Catal. A Gen..

[20] Nayak S., Pradhan A.C., Parida K.M. (2018). Topotactic Transformation of Solvated MgCr-LDH Nanosheets to Highly Efficient Porous MgO/MgCr2O4 Nanocomposite for Photocatalytic H2 Evolution. Inorg. Chem..

[21] Liang S.Y., Ren W.W., Lin W.X., Zou L.C., Cui X.P., Chen J.F. (2020). In-Situ Preparation of MgCr-LDH Nano-Layer on MAO Coating of Mg Alloy and Its Anti-Corrosion Mechanism. Rare Metal Mater. Eng..

[22] Gunawan P., Xu R. (2009). Lanthanide-Doped Layered Double Hydroxides Intercalated with Sensitizing Anions: Efficient Energy Transfer between Host and Guest Layers. J. Phys. Chem. C.

[23] Domínguez M., Pérez-Bernal M.E., Ruano-Casero R.J., Barriga C., Rives V., Ferreira R.A.S., Carlos L.D., Rocha J. (2011). Multiwavelength Luminescence in Lanthanide-Doped Hydrocalumite and Mayenite. Chem. Mater..

[24] Posati T., Costantino F., Latterini L., Nocchetti M., Paolantoni M., Tarpani L. (2012). New Insights on the Incorporation of Lanthanide Ions into Nanosized Layered Double Hydroxides. Inorg. Chem..

[25] Zhang Z., Chen G., Liu J. (2014). Tunable photoluminescence of europium-doped layered double hydroxides intercalated by coumarin-3-carboxylate. RSC Adv..

[26] Gao X., Lei L., Kang L., Wang Y., Lian Y., Jiang K. (2014). Synthesis, characterization and optical properties of a red organic–inorganic phosphor based on terephthalate intercalated Zn/Al/Eu layered double hydroxide. J. Alloys Compd..

[27] Vicente P., Pérez-Bernal M.E., Ruano-Casero R.J., Ananias D., Almeida Paz F.A., Rocha J., Rives V. (2016). Luminescence properties of lanthanide-containing layered double hydroxides. Micropor. Mesopor. Mater..

[28] Smalenskaite A., Salak A.N., Ferreira M.G.S., Skaudzius R., Kareiva A. (2018). Sol-gel synthesis and characterization of hybrid inorganic-organic Tb(III)-terephthalate containing layered double hydroxides. Opt. Mater..

[29] Smalenskaite A., Salak A.N., Kareiva A. (2018). Induced neodymium luminescence in sol-gel derived layered double hydroxides. Mendeleev Commun..

[30] Smalenskaite A., Pavasaryte L., Yang T.C.K., Kareiva A. (2019). Undoped and Eu3+ Doped Magnesium-Aluminium Layered Double Hydroxides: Peculiarities of Intercalation of Organic Anions and Investigation of Luminescence Properties. Materials.

[31] Shirotori M., Nishimura S., Ebitani K. (2016). Genesis of a bi-functional acid–base site on a Cr-supported layered double hydroxide catalyst surface for one-pot synthesis of furfurals from xylose with a solid acid catalyst. Catal. Sci. Technol..

[32] Melo F., Morlanés N. (2008). Study of the composition of ternary mixed oxides: Use of these materials on a hydrogen production process. Catal. Today.

[33] Rodriguez-Rivas F., Pastor A., de Miguel G., Cruz-Yusta M., Pavlovic I., Sánchez L. (2020). Cr3+ substituted Zn-Al layered double hydroxides as UV–Vis light photocatalysts for NO gas removal from the urban environment. Sci. Total Environ..

[34] Correcher V., Garcia-Guinea J. (2018). Cathodo- and photoluminescence emission of a natural Mg-Cr carbonate layered double hydroxide. Appl. Clay Sci..

[35] Ishikawa K., Garskaite E., Kareiva A. (2020). Sol–gel synthesis of calcium phosphate-based biomaterials-A review of environmentally benign, simple, and effective synthesis routes. J. Sol-Gel Sci. Technol..

[36] Karoblis D., Zarkov A., Mazeika K., Baltrunas D., Niaura G., Beganskiene A., Kareiva A. (2020). Sol-gel synthesis, structural, morphological and magnetic properties of BaTiO3–BiMnO3 solid solutions. Ceram. Int..

[37] Grigorjevaite J., Janulevicius M., Kruopyte A., Ezerskyte E., Vargalis R., Sakirzanovas S., Katelnikovas A. (2020). Synthesis and optical properties of efficient orange emitting GdB5O9:Sm3+ phosphors. J. Sol-Gel Sci. Technol..

[38] Pakalniskis A., Marsalka A., Raudonis R., Balevicius V., Zarkov A., Skaudzius R., Kareiva A. (2021). Sol-gel synthesis and study of praseodymium substitution effects in yttrium aluminium garnet Y3-xPrxAl5O12. Opt. Mater..

[39] Jitianu M., Zaharescu M., Bãlãsoiu M., Jitianu A. (2003). The Sol-Gel Route in Synthesis of Cr(III)-Containing Clays. Comparison Between Mg-Cr and Ni-Cr Anionic Clays. J. Sol-Gel Sci. Technol..

[40] Saikia P., Gautam A., Goswamee R.L. (2016). Synthesis of nanohybrid alcogels of SiO2 and Ni–Cr/Mg–Cr–LDH: Study of their rheological and dip coating properties. RSC Adv..

[41] Valeikiene L., Grigoraviciute-Puroniene I., Kareiva A. (2020). Alkaline earth metal substitution effects in sol-gel–derived mixed metal oxides and Mg2-xMx/Al1 (M = Ca, Sr, Ba)–layered double hydroxides. J. Austral. Ceram. Soc..

[42] Shannon R.D. (1976). Revised Effective Ionic Radii and Systematic Studies of Interatomic Distances in Halides and Chalcogenides. Acta Crystallogr..

[43] Smalenskaite A., Sen S., Salak A.N., Ferreira M.G.S., Skaudzius R., Katelnikovas A., Kareiva A. (2016). Sol-Gel Synthesis and Characterization of Non-Substituted and Europium-Substituted Layered Double Hydroxides Mg3/Al1-xEux. Current Inorg. Chem..

[44] Blasse G., Grabmaier B.C. (1994). Luminescent Materials.

[45] Yen W.M., Shionoya S., Yamamoto H. (2007). Fundamentals of Phosphors.

[46] Lahoz F., Martín I.R., Méndez-Ramos J., Núñez P. (2004). Dopant distribution in a Tm3+-Yb3+ codoped silica based glass ceramic: An infrared-laser induced upconversion study. J. Chem. Phys..

[47] Zhu L.L., Hao C., Wang X.H., Guo Y.N. (2020). Fluffy Cotton-Like GO/Zn-Co-Ni Layered Double Hydroxides Form from a Sacrificed Template GO/ZIF-8 for High Performance Asymmetric Supercapacitors. ACS Sustain. Chem. Eng..

